# Ethics and the UN Sustainable Development Goals: The Case for Comprehensive Engineering: Commentary on “Using Student Engagement to Relocate Ethics to the Core of the Engineering Curriculum”

**DOI:** 10.1007/s11948-016-9862-2

**Published:** 2016-12-27

**Authors:** Jeroen van den Hoven

**Affiliations:** Delft University of Technology, Delft, The Netherlands

**Keywords:** Comprehensive engineering, Responsible research and innovation, Engineering ethics, Sustainable development, Sustainable development goals, Global systems science

## Abstract

In the twenty-first century, the urgent problems the world is facing (the UN Sustainable Development Goals) are increasingly related to vast and intricate ‘systems of systems’, which comprise both socio-technical and eco-systems. In order for engineers to adequately and responsibly respond to these problems, they cannot focus on only one technical or any other aspect in isolation, but must adopt a wider and multidisciplinary perspective of these systems, including an ethical and social perspective. Engineering curricula should therefore focus on what we call ‘comprehensive engineering’. Comprehensive engineering implies ethical coherence, consilience of scientific disciplines, and cooperation between parties.

In her article included in this volume “Using student engagement to relocate ethics to the core of the engineering curriculum” Mary Sunderland (2013) discusses an insightful approach to the ethics curriculum for engineers and applied scientists. Following her suggestion, pursuit of the ideal of comprehensiveness should be central to thinking about the future of engineering and engineering ethics alike, and is the most promising direction for the further development of the ethics curriculum. Recent decades have made particularly clear that the problems of the world in the twenty-first century, as they are, for example, expressed in the internationally endorsed *Sustainable Development Goals* (United Nations 2015a), are problems concerning vast “systems of systems”, comprising both socio-technical systems and eco-systems. If the design and management of these complex and elaborate systems are wrong, there is a risk of tragedies of the commons, large scale catastrophes, major social and political instabilities, and individual suffering. If, on the other hand, these are done reasonably well, there is a chance of systematically improving the conditions for sustainable human flourishing.

## UN Sustainable Development Goals

The UN Sustainable Development Goals (SDGs) form a broadly endorsed catalogue of the major challenges that mankind must come to grips with in order to make it to the next century. Building on a set of UN Resolutions [e.g., *The World We Want* (United Nations 2015b)], the UN General Assembly in September 2015 signed a program into action entitled “Transforming our world: the 2030 Agenda for Sustainable Development” (United Nations 2015c). The SDGs now form an intergovernmental set of 17 aspirational goals, broken down into 169 specific targets. These include, but are not limited to, ‘ending poverty and hunger’, ‘improving health and education’, ‘making cities more sustainable’, ‘combating climate change,’ and ‘protecting oceans and forests’.

It does not make much sense to pursue these goals individually or to temporarily disregard one (e.g., women’s education or climate change) in order to focus on another (e.g., poverty reduction or rule of law and stronger institutions). Problems arising in regard to these interlocked and overlapping goals require integral thinking and ‘global systems science’ solutions (Helbing 2013). The required sense of comprehensive systems implies the study of natural systems (e.g., rainforest, reef, the atmosphere and the arctic). It equally points to the study of large scale socio-technical systems and infrastructures (e.g., maritime, air and land transport systems, energy systems, urban regions, and industrial production). Finally it points to a better understanding of human behaviour and institutional, economic, and moral aspects of human action.

On the basis of more than two decades of teaching and research in engineering ethics (Davis 2005; Mitcham and Englehardt 2016; van de Poel and Royakkers 2011), value sensitive design and responsible research and innovation (van den Hoven 2013), and the philosophy of technology (Franssen et al. 2013), one can safely conclude that the ethics of engineering and applied science in the twenty-first century requires efforts from multiple disciplines, and requires the perspectives of direct and indirect stakeholders, public and private partners, and representatives of industry, education and government alike (Sunderland et al. 2014). In order to usefully contribute to solutions to the SDGs, the net needs to be cast widely, both in terms of knowledge and skills, and in terms of involvement of parties. This is also argued in a recent collection of essays *Engineering Ethics for a Globalized World* (Murphy et al. 2015).

It is extremely unlikely that solutions to these problems can be found in one specialized branch of knowledge or in one discipline, but it is also clear that thinking about them involves many interrelated ethical issues that one cannot come to grips with and properly study in isolation from other ethical issues. Climate Change, for example, requires us to look at moral motivation, the logic of public goods dilemmas and tragedies of the common, the moral limits of nudging and choice modelling techniques, the limits of stimulating responsible innovation in energy systems by means of financial incentives, the ways to deal with moral compromise that may facilitate political breakthroughs in climate negotiations, designing fiscal measures for industry, thinking about discount rates and future generations in economic models, criteria for fair distribution of risks, and new regulation and new governance models appropriate for grids that allow for decentralized and distributed energy production. At the end of the day, society needs to be able to justify its actions, policies and plans regarding climate change. Studies of water and food problems have increased awareness of the Energy–Food–Water Nexus. New mobility and transport systems with autonomous components and self-driving vehicles may require new forms of insurance, liability, regulation, governance, road infrastructure, monitoring and inspection. Industry 4.0 opens up a range of questions concerning technical unemployment, safety, security, privacy, and responsibility. All of these questions raise issues of the fair distribution of risk, benefits and harms, and all raise further questions about apportioning responsibilities.

As the prominent philosopher of law Jeremy Waldron puts it in his comments on the work of the *Global Citizen Commission* (on which he served together with philosopher and Nobel Prize winner in Economics Amartya Sen, and Mohammed Elbaradei [former Secretary General of the International Atomic Agency] and under the chairmanship of former UK prime minister Gordon Brown): “as the world becomes increasingly interconnected, the importance of (…) global-minded ethical considerations is becoming ever clearer” (Waldron 2016). The philosopher Ronald Dworkin observed, in “A New Philosophy for International Law,” what is needed is an “…international legislative body with sufficient jurisdiction to solve the grave coordination problems that every nation now confronts. We are already seized by devastating prisoners’ dilemmas: about terrorism, climate change, Internet communication, and economic policy” (Dworkin 2013, p. 27). Dworkin argues that global society needs an entirely different form of international organization so that it can “attack those problems through *comprehensive* global legislation” (Dworkin 2013, p. 27, emphasis added).

The discoverer of the quark, Nobel Prize winner, and co-founder of the Stanford Santa Fe institute, Murray Gell-Mann spoke in his book *The Quark and the Jaguar* about the much needed synthesis of two cognitive styles, the Dionysian (passion, synthesis, intuition) and the Apollonian (analysis, rationality) in the so-called “Odyssean” cognitive style that aims at overcoming this bifurcation by means of integrative and comprehensive thinking: “(…) the crude study of world problems including all the relevant aspects, not only environmental, demographic and economic, but also social, political, military, diplomatic and ideological” (Gell-Mann 1994, p. xii). He continues “Those who study complex adaptive systems are beginning to find some general principles that underlie all such systems, and seeking out those principles requires intensive discussions and collaboration among specialists in a great many fields. … integration of those fields is urgently needed. Important contributions are made by the handful of scholars and scientists who are transforming themselves from specialists into students of simplicity and complexity or of complex adaptive systems in general” (Gell-Mann 1994, p. xiii).

Michael Dertouzos, the former Director of the Massachusetts Institute of Technology (MIT) Laboratory for Computer Science, observed more than a decade ago that the involvement of the social sciences is no longer enough to do a good job in engineering and technology. The prominent role of technology in global societies calls for the integration of engineering, social sciences, and the humanities (ethics and philosophy). This requires an extra effort since “Universities isolated technologists from humanists in watertight compartments across campus from each other. … If we allow this trend to continue, our problem will increase and we will miss the prospect of being better off in the biggest possible sense of being human” (Dertouzos 2002, p. 215). According to Dertouzos, twenty-first century problems cannot be solved by a purely scientific approach, nor by an approach that has its sole basis in the humanities: “Technology will be as important … as humanistic ideals were and will continue to be. Keeping the technologists separated from the humanists will keep us from discovering these new territories. … If we remain fragmented, we’ll be unable to fulfil our full human potential, because we will be running on only some of our cylinders” (Dertouzos 2002, p. 216).

In her fictional dystopian account *The Collapse of Western Civilization*—cast in the form of a report of an historian looking back from the future (after a global deluge that has flooded vast and densely populated coastal areas and deltas of the world)—the Harvard historian of science Naomi Oreskes identifies some of the factors that cause people to ignore the nature of major global problems—such as climate change and sea level rising. The root cause for this inadequacy lies, according to Oreskes, in a number of epistemic flaws. First of all, humans are beset by a form of reductionism that deals with components and parts of complex problems in isolation. Furthermore, there is an obsession with tractability which is valuable in physics and medical diagnostics, but is not very helpful in climate science, disaster management, economics, transportation or energy. In these areas decision makers need to be able to think comprehensively in terms of systems of systems. But even that needs to be done in a sufficiently rich way, because otherwise policy makers tend to ignore “the idea of system science, complexity science, and, most pertinent to our purposes here (i.e., the discussion of climate change) earth system science, but these so-called holistic approaches still focused almost entirely on natural systems, omitting from consideration the social components. Yet in many cases, the social components were the dominant system drivers” (Oreskes 2014, p. 15).

Pope Francis has made observations to this very same point in his encyclical writing on climate change. He calls for an integral approach to ecological issues. Climate change calls for “a *comprehensive* approach” (Bergoglio 2015, Sect. 135), and stipulates that it “is essential to seek *comprehensive solutions*” (Bergolio 2015, Sect. 139):

…concerning solving the more complex problems of today’s world, particularly those regarding the environment and the poor; these problems cannot be dealt with from a single perspective or from a single set of interests. A science which would offer solutions to the great issues would necessarily have to take into account the data generated by other fields of knowledge, including philosophy and social ethics; but this is a difficult habit to acquire today (Bergolio 2015, Sect. 110).

(…)

Since everything is closely interrelated, and today’s problems call for a vision capable of taking into account every aspect of the global crisis, I suggest that we now consider some elements of an integral ecology, one which clearly respects its human and social dimensions.” (…) “It is essential to seek comprehensive solutions which consider the interactions within natural systems themselves and with social systems (Bergolio 2015, Sect. 120).

What Gell-Mann, Pope Francis, Oreskes and Dertouzos (and indirectly the legal scholars Dworkin and Waldron) call for is what can be called ‘comprehensive engineering’,^1^ i.e., a form of complex systems engineering, design, regulation and management of socio-technical systems that accommodates the hybrid and strongly coupled nature of the (socio-technical-eco) systems that make up the world in the twenty-first century; their dynamics, complexity and moral, social and technical aspects.

The idea of comprehensive engineering should be central to thinking about ethics and engineering and the global challenges as codified in the UN SDGs. I think this requires recognizing and distinguishing three aspects or dimensions of the idea of comprehensiveness: (1) consilience (an epistemic aspect), (2) coherence (a moral aspect), and (3) collaboration (a social aspect) of thinking about solutions.

### Consilience

Consilience is the idea that reality is one and intelligible as such. The object of study is basically one world that is studied with different methods and tools from different disciplinary perspectives. For example, whether one measures the distance between the pyramids of Gizeh by means of a rope, satellites or infrared, one still expects to get more or less the same answer to the question “how far apart are they?”. The same applies to the combination of carbon-dating, geological data, dendro-chronological readings, and archeological data: one expects them to converge across archeological studies. In climate change, the situation is more complicated. The units of analysis here are, as pointed out above, ‘systems of systems’ or complex adaptive systems that need to be analyzed, managed and (re-)designed. However, they typically comprise human beings with mental states who act, guided by incentive structures, laws, institutions and procedures, and moral reasons, which all interact—often in ways ill-understood—with technical components, processes, software and hardware, and infrastructures. The presence of human individuals and collective agents, with mental states, such as beliefs, hopes, fears, and expectations, makes it more difficult to (quantitatively) model them and to integrate understanding of them with understanding of the physical and natural parts of the systems. John Searle has pointed out that this may require extra effort, but it is not necessarily a show stopper for scientific understanding: ontological subjectivity does not preclude epistemic objectivity (Searle 2002). But it does imply that what is needed are all the disciplines that have the best track record in shedding light on these heterogeneous systems, that obey different laws and different logics. Behavioral economics, evolutionary game theory, ethology, mathematics, modal logic, and experimental ethics are required here.

Traditionally, the notion of consilience has received a reductionist interpretation. According to this interpretation, propositions about human behavior reduce to propositions about brain states and states of the world (including the human body), which in turn reduce to propositions about physiology and chemistry, which reduce to statements about physics and further down to elementary particle physics. This is what philosopher of mind and language Jerry Fodor has called ‘vertical consilience.’ The causal pathways extend deep into the building blocks of life and matter. This is to be contrasted with ‘horizontal consilience’, which refers to coherence of disciplinary perspectives. Horizontal consilience is what makes interdisciplinary studies in Climate Change a feasible ideal for policy makers. The idea of consilience was revived by the Harvard entomologist E.O. Wilson. He gave it a strong reductionist reading. Fodor reviewed *Consilience* in the *London Review of Books* arguing that Wilson “fails to notice the difference between what one might call a vertical and horizontal consilience” (Fodor 1998). The former provides the paradigms for the unification program, the idea that all science is basically one and all scientific claims are grounded in claims about the physical world, a thesis defended by advocates of logical positivism. Horizontal consilience gives rise to hyphenated disciplines, such as socio-biology, physical anthropology, and psycho-linguistics. Horizontal consilience just implies that “The web of causal explanation is extended; but side ways, not up and down” (Fodor 1998). Thus, the study of socio-technical systems of systems that figure prominently in climate change, energy transition, cyber security and transport systems, also involves webs of explanation that extend sideways.

### Coherence

Another important ingredient of comprehensive engineering pertains to the fact that the ethical issues themselves are interconnected and often cannot be dealt with in isolation. This is the case because moral thinking tracks the real world in its complexity and unity and supervenes on its myriad causal interconnections. Moral issues are considered to ‘supervene’ on the non-moral world, which implies that there can be no change in the moral world without a corresponding change in the non-moral world. Ethical issues in the climate change debate—as political philosopher Simon Caney suggests^2^—cannot be given an adequate treatment when considered in isolation from other ethical issues: “One important issue when considering the normative issues raised by climate change is whether it is best to treat these issues in ‘isolation’ from other normative issues (and thus to construct an account of climate justice that brackets out other issues such as poverty, development, trade, migration and so on (‘isolationism’); or whether it is best to treat the ethical issues raised by climate change in conjunction with a more general account of justice (‘integrationism’). In my research, I have defended what I term an ‘integrationist’ approach in a number of places” (See also Caney 2012, p. 259). This integrationism also holds for the SDGs. Engineers are not pursuing solutions to one goal at a time, but need to move them *en bloc, not one at the expense of another,* and therefore require an integrated approach.

Similar claims could be made about ethical issues concerning digital technology. First, privacy and intellectual property are concerned with preventing access to information, whereas transparency, accountability and equity concern the provision of access to information (sometimes the very same information that is protected on privacy grounds). Furthermore, democratic governance and fair regulation of the internet may pertain to both issues. Solutions to these problems are interrelated and arguably cannot be dealt with in isolation.

### Cooperation

Philosopher Christine Korsgaard has pointed out that “one of the most important attributes of humanity is our nearly bottomless capacity for conferring value on most anything. It is not because of our shared values that we should accord consideration to one another but because of our shared capacity for conferring value” (Korsgaard 2005, p. 73). Different individuals and groups represent different world views, have access to different information and identify different ultimate goals. They highlight different features of the world and situations as salient and relevant. Cooperation and participation and involvement of a diversity of people and a broad range of perspectives is both epistemically fortuitous and morally required for legitimacy of decisions and respect for persons.

In the twenty-first century engineers who want to address societal problems of the type we have discussed here and want to address the ethical and societal aspects of technology adequately, need to proceed *comprehensively*. They will leave out perspectives, parties and disciplines at the expense of adequate and sustainable solutions. It seems fair to say that the pursuit of consilience, coherence and cooperation is implied in this ideal of comprehension.

## Comprehensive engineering, ethics and the engineering curriculum

The applied sciences and engineering, the social sciences, and the humanities are all of crucial importance in comprehensively addressing the world’s grand challenges as expressed in the United Nations SDGs. Both engineering ethics and engineering curricula should therefore not focus solely on the micro level and on innovations in isolation. Rather, ecosystems and socio-technical systems, and the systems of systems comprising them, will together often form the appropriate level of analysis. Whereas many programs in engineering and engineering ethics in the world are aiming to integrate engineering and the social sciences, or engineering and ethics, only a very few programs investigate the normative and ethical aspects in combination with engineering and the social sciences.

Questions about how to shape, design, steer, and govern these ensembles of complex socio-technological and natural systems responsibly are among the hardest questions that confront humanity. The engineers of the future must be prepared, encouraged and supported to rise to the occasion, becoming *comprehensive engineers,* who aim for ethical coherence, consilience of disciplines, and cooperation between parties.

## Footnotes

1 See a short video for an introduction and application of the idea of Comprehensive Engineering at www.tpm.tudelft.nl.

2 Simon Caney, Methodological Issues, http://simoncaney.weebly.com/methodological-issues.html (accessed December 14, 2016).

## References

[CR1] Bergoglio, J. M. [Pope Francis]. (2015). *Laudato si*. http://w2.vatican.va/content/francesco/en/encyclicals/documents/papa-francesco_20150524_enciclica-laudato-si.html. Accessed November 9, 2016.

[CR2] Caney S (2012). Just emissions. Philosophy & Public Affairs.

[CR3] Davis M (2005). Engineering ethics.

[CR4] Dertouzos M (2002). The unfinished revolution.

[CR5] Dworkin M (2013). A new philosophy for international law. Philosophy & Public Affairs.

[CR6] Fodor, J. (1998). *Look*! http://www.lrb.co.uk/v20/n21/jerry-fodor/look. Accessed November 9, 2016.

[CR7] Franssen, M., Lokhorst, G. J., & van de Poel, I. (2013). Philosophy of technology. In *Stanford encyclopedia of philosophy* http://plato.stanford.edu/entries/technology/. Accessed November 9, 2016.

[CR8] Gell-Mann M (1994). The quark and the jaguar.

[CR9] Helbing D (2013). Globally networked risks and how to respond. Nature.

[CR10] Korsgaard C, Raz J (2005). The dependence of value on humanity. The practice of value.

[CR11] Mitcham C, Englehardt EE (2016). Ethics across the curriculum: prospects for broader (and Deeper) teaching and learning in research and engineering ethics. Science and Engineering Ethics.

[CR12] Murphy, C., Gardoni, P., Bashir, H., Harris, C. E., Jr. & Masad, E. (Eds.). (2015). Engineering ethics for a globalized world. *Philosophy of Engineering and Technology.* doi:10.1007/978-3-319-18260-5.

[CR13] Oreskes N (2014). The collapse of western civilization.

[CR14] Searle J, Searle J (2002). Consciousness. Consciousness and language.

[CR15] Sunderland ME (2013). Using student engagement to relocate ethics to the core of the engineering curriculum. Science and Engineering Ethics.

[CR16] Sunderland ME, Taebi B, Carson C, Kastenberg WE (2014). Teaching global perspectives: Engineering ethics across international and academic borders. Journal of Responsible Innovation.

[CR17] United Nations. (2015a). *Sustainable development goals*. http://sustainabledevelopment.un.org/?menu=1300. Accessed November 9, 2016.

[CR18] United Nations. (2015b). *The world that we want*. http://www.un.org/millenniumgoals/pdf/UNDG%202nd%20dialogues.pdf. Accessed November 9, 2016.

[CR19] United Nations. (2015c). *Transforming our world: The 2030 agenda for sustainable development*. http://sustainabledevelopment.un.org/post2015/transformingourworld. Accessed November 9, 2016.

[CR20] van de Poel I, Royakkers L (2011). Ethics, technology and engineering.

[CR21] van den Hoven J, Innovation Responsible (2013). Responsible innovation and value sensitive design. Richard owen.

[CR22] Waldron, J. (2016). *Why we all need to begin thinking like global citizens*. WEForum. http://www.weforum.org/agenda/2016/04/why-we-all-need-to-begin-thinking-like-global-citizens. Accessed November 9, 2016.

